# Impaired cerebral compensatory reserve is associated with admission imaging characteristics of diffuse insult in traumatic brain injury

**DOI:** 10.1007/s00701-018-3681-y

**Published:** 2018-09-24

**Authors:** Frederick A. Zeiler, Dong-Joo Kim, Manuel Cabeleira, Leanne Calviello, Peter Smielewski, Marek Czosnyka

**Affiliations:** 1 Division of Anaesthesia, Addenbrooke’s Hospital, University of Cambridge, Cambridge, UK; 2 Section of Surgery, Rady Faculty of Health Sciences, University of Manitoba, Winnipeg, MB Canada; 3 Clinician Investigator Program, Rady Faculty of Health Science, University of Manitoba, Winnipeg, Canada; 4 Department of Brain and Cognitive Engineering, Korea University, Seoul, South Korea; 5 Section of Brain Physics, Division of Neurosurgery, Department of Clinical Neurosciences, Addenbrooke’s Hospital, University of Cambridge, Cambridge, CB2 0QQ UK; 6 Institute of Electronic Systems, Warsaw University of Technology, Warsaw, Poland

**Keywords:** Compensatory reserve, Diffuse injury, Imaging, Monitoring, TBI

## Abstract

**Background:**

Continuous assessment of cerebral compensatory reserve is possible using the moving correlation between pulse amplitude of intra-cranial pressure (AMP) and intra-cranial pressure (ICP), called RAP. Little is known about the behavior and associations of this index in adult traumatic brain injury (TBI). The goal of this study is to evaluate the association between admission cerebral imaging findings and RAP over the course of the acute intensive care unit stay.

**Methods:**

We retrospectively reviewed 358 adult TBI patients admitted to the Addenbrooke’s Hospital, University of Cambridge, from March 2005 to December 2016. Only non-craniectomy patients were studied. Using archived high frequency physiologic signals, RAP was derived and analyzed over the first 48 h and first 10 days of recording in each patient, using grand mean, percentage of time above various thresholds, and integrated area under the curve (AUC) of RAP over time. Associations between these values and admission computed tomography (CT) injury characteristics were evaluated.

**Results:**

The integrated AUC, based on various thresholds of RAP, was statistically associated with admission CT markers of diffuse TBI and cerebral edema. Admission CT findings of cortical gyral effacement, lateral ventricle compression, diffuse cortical subarachnoid hemorrhage (SAH), thickness of cortical SAH, presence of bilateral contusions, and subcortical diffuse axonal injury (DAI) were all associated with AUC of RAP over time. Joncheere-Terpstra testing indicated a statistically significant increase in mean RAP AUC across ordinal categories of the abovementioned associated CT findings.

**Conclusions:**

RAP is associated with cerebral CT injury patterns of diffuse injury and edema, providing some confirmation of its potential measurement of cerebral compensatory reserve in TBI.

**Electronic supplementary material:**

The online version of this article (10.1007/s00701-018-3681-y) contains supplementary material, which is available to authorized users.

## Introduction

From a clinical perspective, quantitative knowledge of cerebral compensatory reserve/compliance can prove useful in the management of traumatic brain injury (TBI) patients. Such monitoring can provide insight into future expectations of refractory intra-cranial pressure (ICP) control, cerebral herniation syndromes, and subsequent clinical deterioration. Furthermore, changes in compensatory reserve may precede, or provide an “early warning,” for subsequent lesion progression (such as contusion progression), or new lesion formation (such as a cerebral infarct).

Previous attempts at directly measuring cerebral compliance/compensatory reserve have highlighted the importance of the relationship between pulse amplitude and ICP, among other cerebral blood volume (CBV) measures [1, 26]. These studies outlined the strong linear relations between pulse amplitude of ICP and ICP itself. Furthermore, other techniques for compliance analysis have been attempted in the past including, but not limited to ICP slope analysis [12], ICP pulse amplitude in isolation [6, 8, 12], assessing ICP waveform morphology [11, 13, 14, 24], TCD-based pulsatility indices and CBV estimates [2, 5, 10], and magnetic resonance imaging (MRI)-based elastography [3, 16]. All of these previous measures suffer from complexity in signal analytic techniques and intermittent or semi-intermittent data capture (i.e., TCD and MRI elastography).

More recently, the moving correlation coefficient between slow-waves of pulse amplitude of ICP (AMP) and ICP, called RAP (R, correlation; A, AMP; P, ICP), has been previously proposed as a surrogate measure of compensatory reserve [4, 15]. The majority of the clinical literature to date focuses on the application of this particular index in monitoring hydrocephalus patients and assessing compensatory reserve during cerebrospinal fluid (CSF) infusion studies [7, 15]. Though a recent study evaluated compensatory reserve weighted ICP measurements, where weighted ICP = ICP × (1-RAP), which demonstrated that weighted ICP measures was associated with mortality in adult TBI [4].

Difficulties regarding the interpretation of this index have likely limited its widespread use. RAP values near zero, or negative, can indicate both preserved and impaired cerebral compensatory reserve, depending on which aspect of the inverse parabolic relationship is being observed [9, 27]. Plots of RAP vs ICP or cerebral perfusion pressure (CPP) demonstrate an inverse parabolic relationship, with moderate/severe impairment of compensatory reserve indicated by positive values of RAP. However, values near zero can indicate either (a) preserve compensatory reserve during episodes of low ICP or (b) extreme exhaustion of compensatory reserve during sustain elevations in ICP. This relationship has been detailed in previous publications, including a recent work in adult TBI patients [27].

There is a paucity of literature evaluating the associations between RAP and clinically relevant variables, particularly in TBI where this type of compensatory reserve monitoring may prove valuable. Of particular interest in TBI is the association between RAP and cerebral imaging characteristics of diffuse injury/edema, as well as the association with global patient outcome. The goal of this study in adult TBI patients is to evaluate the association between RAP, during both the first 48 h and first 10 days of physiologic recording and admission brain CT characteristics of injury, providing some of the first evidence in support of RAP as a measure of cerebral compensatory reserve. This is the first study to assess these relationships.

## Methods

### Patient population

This patient population has been previously described in studies pertaining to the definition of a new cerebrovascular reactivity index [27], characterizing critical thresholds of ICP derived reactivity indices [29], and the assessment of imaging correlates to impaired cerebrovascular reactivity in adult TBI [28]. This population has a detailed admission CT injury characteristic database that was created for the previous work on cerebrovascular reactivity [28]. This data is unique and provided the ability to evaluate the link between RAP and admission imaging characteristics. Hence, we retrospectively reviewed this population for the purpose of this study.

This study was conducted as a retrospective analysis of a prospectively maintained database cohort, in which high frequency clinical neuromonitoring data had been archived. Monitoring of brain modalities was conducted as a part of standard neurosciences critical care unit (NCCU) patient care using an anonymized database of physiological monitoring variables in neurocritical care. Data on age, injury severity, and clinical status at hospital discharge were recorded at the time of monitoring on this database, and no attempt was made to re-access clinical records for additional information. Since all data was extracted from the hospital records and fully anonymized, no data on patient identifiers were available, and need for formal patient or proxy consent was waived. Within our institution, patient data may be collected with waiver of formal consent, as long as it remains fully anonymized, with no method of tracing this back to an individual patient. Patient physiologic, demographic, and outcome data were collected by the clinicians involved with patient care, and subsequently recorded in an anonymous format. This anonymous data is then provided for future research purposes. Such data curation remains within compliance for research integrity as outlined in the UK Department of Health - Governance Arrangements for Research Ethics Committees (GAfREC), September 2011 guidelines, “Conclusions” section [25].

As mentioned in our previous studies, all patients (*n* = 358) were admitted to the Neurosciences and Trauma Critical Care Unit (NCCU) at the Cambridge University Hospitals NHS Foundation Trust (CUH), during the period of March 2005 to December 2016. Patients with primary or secondary decompressive craniectomy were excluded, due to its known impact on ICP and ICP-derived vascular reactivity indices. All patients had archived high frequency physiologic signals stored within the signal database at the Addenbrooke’s Hospital. Patients suffered either moderate to severe TBI or mild TBI and subsequently deteriorated to a point where they required intra-cranial monitoring, sedation, and mechanical ventilation. Given this, patients received ICP monitor placement at varying times post-injury based on clinical need. Treatment received during the recording periods included standard ICP-directed therapy, with an ICP goal of less than 20 mmHg and CPP goal of greater than 60 mmHg.

### Admission cranial CT injury characteristics

All admission brain CT injury data was collected as part of a previous study, evaluating the link between admission CT characteristics and impaired cerebrovascular reactivity in TBI [28]. This database of injury characteristics was retrospectively accessed for the purpose of this study. All scans were assessed by a qualified specialist neurosurgeon for a variety of injury characteristics.

Each CT scan had a detailed assessment of IC injury, as measured by standard CT scoring systems (Marshall, Rotterdam, Helsinki, and Stockholm). In addition, a comprehensive CT lesion/characteristic database was available for each admission CT scan. This database has been detailed in the previous publication by Zeiler et al. [28]. This IC injury database consisted of

1. Continuous variables: midline shift (MLS), in millimeters (mm); largest lesion volume, in milliliters (mL); number of contusions; number of diffuse axonal injury (DAI) lesions; and total contusion volume, in mL.

MLS was measured on the admission CT using the distance of the septum pellucidum from boney midline (derived from the line connecting the crista gallae to the inion), at the level of the foramen of Monro. Volumes were calculated using the A × B × C / 2 method [17] for contusions and extra-axial hematomas from the admission CT scans, where *A* is the maximal antero-posterior length in centimeters (cm), *B* is the maximal thickness in cm, and *C* is the number of 1-cm CT slices (where slices with ≥ 75% area of hemorrhage, counts as 1 slice; slices with 25–75% area of hemorrhage, counts as 0.5 slices; slices with < 25% area of hemorrhage, counts as 0 slices) [17]. Volumes for each contusion were calculated individually.

2. Ordinal characteristics:
  - Basal cistern compression (none, compressed, complete)
  - Lateral ventricle compression (none, compressed, complete)
  - Convexity gyral compression (none, compressed, complete)
  - Fourth ventricle compression (none, compression, complete)
  - Convexity traumatic subarachnoid hemorrhage (tSAH) extent (none, visible in gyri, > 90% of bilateral hemispheric coverage)
  - Convexity tSAH maximal thickness (none, 1–5 mm, > 5 mm)
  - Cisternal tSAH extent (none, visible, completely filled)
3. Binary characteristics:
  - Basal cistern compression (none, any compression amount)
  - Extreme basal cistern compression (none, complete effacement)
  - Lateral ventricle compression (none, any compression)
  - Fourth ventricle compression (none, any compression)
  - Extreme fourth ventricle compression (none, complete effacement)
  - Tonsillar decent (no, yes), J. any lesion > 25 mL (no, yes)
  - Convexity subdural hematoma (SDH) (no, yes)
  - Tentorial SDH (no, yes)
  - Falcine SDH (no, yes)
  - Bilateral convexity SDH (no, yes)
  - Convexity EDH (no, yes)
  - Bilateral convexity EDH (no, yes)
  - Contusion present (no, yes)
  - Bilateral contusions (no, yes)
  - Intra-ventricular hemorrhage (IVH) (no, yes)
  - Cisternal tSAH (no, yes)
  - Cisternal tSAH completely filled (no, yes)
  - DAI, subcortical (SC) (no, yes)
  - DAI, corpus callosum (CC) (no, yes)
  - DAI, basal ganglia (BG) (no, yes)
  - DAI, brainstem (BS) (no, yes)

### Signal acquisition

Arterial blood pressure (ABP) was obtained through either radial or femoral arterial lines connected to pressure transducers (Baxter Healthcare Corp. CardioVascular Group, Irvine, CA). ICP was acquired via an intra-parenchymal strain gauge probe (Codman ICP MicroSensor; Codman & Shurtleff Inc., Raynham, MA). All signals were recorded using digital data transfer or digitized via an A/D converter (DT9801; Data Translation, Marlboro, MA), where appropriate, sampled at frequency of 50 Hz or higher, using the ICM+ software (Cambridge Enterprise Ltd., Cambridge, UK, http://icmplus.neurosurg.cam.ac.uk). Signal artifacts were removed using both manual and automated methods prior to further processing or analysis.

For the purpose of this study, we only focused mainly on reporting the analysis on the first 10 days of recording. The analysis was also repeated for the first 48 h of recording in order to assess any differences in significant results, though the results are reported in a limited fashion within the body of the manuscript, and the majority of this analysis can be found in Appendix B. The reason for the limited reporting of the first 48-h analysis is related to the uncertainty of the accuracy of onset of data recording, based on our database limitations. It must be re-emphasized that given the heterogeneity of the patients admitted, ICP monitors were placed at various time points post-injury. The exact time frame from injury to placement of the monitor, and thus initiation of signal recording, is not available within the database, and such data is not able to be retrospectively collected given the long time frame over which the patients were admitted and lack of documentation. With that said, the majority of patients likely had ICP monitor placement within 24 to 48 h of injury, but others would have been within approximately the first 5 days depending on clinical condition on admission and neurological deterioration. Thus, when we refer to the time period of monitoring, it is referring to the number of days from the initiation of ICP monitoring, not from injury.

### Signal processing

Post-acquisition processing of the above signals was conducted using ICM+. CPP was determined as CPP = MAP – ICP. AMP was determined by calculating the fundamental Fourier amplitude of the ICP pulse waveforms over a 10-s window, updated every 10 s. Ten second moving averages (updated every 10 s to avoid data overlap) were calculated for all recorded signals: ICP, ABP (which produced MAP), AMP, and CPP.

RAP was determined using the moving Pearson correlation coefficient between 30 consecutive 10-s non-overlapping averages of AMP and ICP. RAP was updated every minute, in keeping with standard calculation methods employed for moving indices in the TBI literature.

### Statistics

Statistics were performed utilizing XLSTAT (Addinsoft, NY, USA; https://www.xlstat.com/en/) add-on package to Microsoft Excel (Microsoft Office 15, Version 16.0.7369.1323) and R statistical software (R Core Team (2016). R: A language and environment for statistical computing. R Foundation for Statistical Computing, Vienna, Austria. URL https://www.R-project.org/). The following R packages were employed: *dplyr*, *tidyverse*, *lubridate*, *Hmisc*, *pROC*, and *flux.* Simple descriptive statistics were employed to outline the overall population demographics. Normality was assessed via the Shapiro-Wilks test. RAP variables (described below) were compared between male and female using the Mann-Whitney *U* test. Correlation with age for RAP variables was assessed via the Pearson correlation coefficient and linear models. For all statistical tests described, alpha was set at 0.05 for significance.

### RAP processing

Minute-by-minute RAP data was further processed using the R statistical software. Given issues in the interpretation of RAP, as eluded to within the introduction, various variables were created from minute-by-minute RAP data across both the first 48 h and first 10 days of recording. These included grand mean per patient over both the entire first 48 h and 10 days of recording, percentage of time above various RAP thresholds, and integrated area under the curve (AUC) for RAP over time (using various baseline thresholds of RAP). Higher mean values for all of these derived RAP variables denote progression in the impairment of cerebral compensatory reserve.

Percentage of time above threshold for RAP was assessed for the following thresholds: 0, + 0.2, + 0.3, + 0.4, + 0.5, + 0.6, and + 0.7. This threshold upper limit of + 0.7 for RAP was based on the population-based error bar plot of RAP vs CPP and RAP vs ICP, seen in our previous population, where the majority of RAP values fall below + 0.7 [27].

**Fig. 1:**
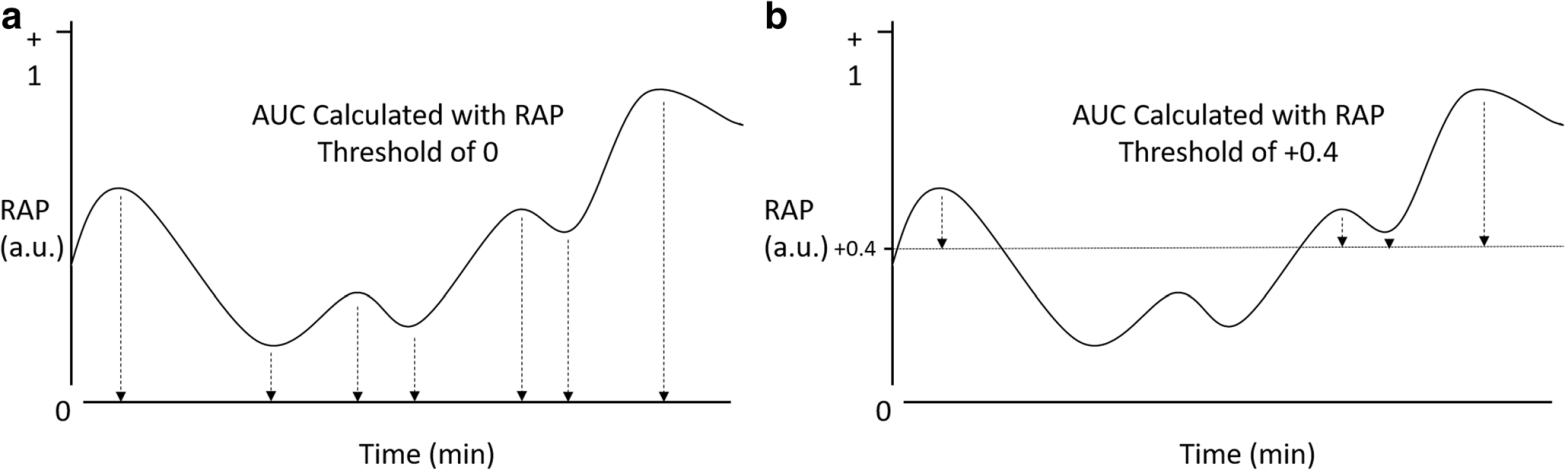
Diagrammatic representation of AUC of RAP over time based on RAP threshold. **a** Displays diagram of RAP over time, with the vertical hashed arrows denoting area under the curve considered for calculation with baseline of 0. **b** Displays diagram of RAP over time, with hashed vertical arrow denoting the area under the curve considered for calculation with a RAP baseline set to + 0.4 (horizontal line). a.u., arbitrary units; AUC, integrated area under the RAP over time curve; min, minutes; ICP, intra-cranial pressure; RAP, correlation between pulse amplitude of ICP (AMP) and ICP

AUC for the RAP was determined for each patient by integrating the RAP signal over time via a sequential linear interpolation method within R using minute-by-minute data. This was conducted over the entire first 48 h and first 10 days of recording for the patient. RAP AUC over time was calculated for RAP thresholds of 0, + 0.2, + 0.3, + 0.4, + 0.5, + 0.6, and + 0.7. Figure 1 provides an overview of area calculation method based on different thresholds of RAP.

### Intra-cranial CT characteristics

Summary statistical details regarding the CT characteristics within the patient population can be found within the appendix section of our previous publication on injury burden and cerebrovascular reactivity [28]. Ordinal CT injury characteristics and CT scoring systems employed in TBI (i.e., Marshall, Rotterdam, Helsinki, and Stockholm grades) were assessed. Statistically significant increase in the various RAP variables (describe in the previous section) across increasing injury severity categories (based on CT findings) was assess via the Joncheere-Terpstra test, with statistically significant increase in RAP variables indicating worsening compensatory reserve with increase in injury severity. Associations between CT-based continuous variables (i.e., midline shift (MLS), total contusion volume, and number of diffuse axonal injury (DAI) lesions) were assessed via the Pearson correlation coefficients and univariate linear models.

Only statistically significant relationships are detailed within the body of the manuscript, with non-significant CT characteristics for the first 10 days and first 48 h of recording briefly listed in Tables 1 and 2, respectively. Details of statistically significant results related to the first 10 days and first 48 h analysis can be found in Appendices A and B, respectively.

**Table 1:** Non-significant admission CT characteristics—first 10 days of recording analysis

Non-significant CT grade systems	Non-significant continuous CT variables
Marshall CT grade	MLS
Rotterdam CT grade	Number of DAI lesions
Helsinki CT grade	Total contusion volume
Stockholm CT grade	
Non-significant ordinal/binary CT variables	
Basal cistern compression (none/any)	Bilateral convexity SDH (no/yes)
Basal cistern compression (none/complete)	Any contusion present (no/yes)
Basal cistern compression (none/compressed/complete effacement)	EDH (no/yes)
Fourth ventricle compression (none/any)	Bilateral EDH (no/yes)
Fourth ventricle compression (none/compressed/complete effacement)	IVH (no/yes)
Fourth ventricle extreme compression (none/completely effaced)	Cisternal tSAH (no/yes)
Lateral ventricle compression (none/mild/complete effacement)	Cisternal tSAH completely filled (no/yes)
Tonsillar descent (no/yes)	Cisternal tSAH extent (none/visible/complete filling)
Convexity SDH (no/yes)	DAI – CC (no/yes)
Tentorial SDH (no/yes)	DAI – BG (no/yes)
Falcine SDH (no/yes)	DAI – BS (no/yes)

*BG*, basal ganglia; *BS*, brain stem; *CC*, corpus callosum; *CT*, computed tomography; *DAI*, diffuse axonal injury; *EDH*, epidural hematoma; *IVH*, intra-ventricular hemorrhage; *MLS*, mid-line shift; *SC*, subcortical; *SDH*, subdural hematoma

**Table 2:** Non-significant admission CT characteristics—first 48 h of recording analysis

Non-significant CT grade systems	Non-significant continuous CT variables
Marshall CT grade	MLS
Rotterdam CT grade	Number of DAI lesions
Helsinki CT grade	Total contusion volume
Stockholm CT grade	
Non-significant ordinal/binary CT variables	
Cortical gyral effacement (none/compressed/complete)	Bilateral convexity SDH (no/yes)
Basal cistern compression (none/any)	Bilateral contusion (no/yes)
Basal cistern compression (none/complete)	Any contusion present (no/yes)
Basal cistern compression (none/compressed/complete effacement)	EDH (no/yes)
Fourth ventricle compression (none/any)	Bilateral EDH (no/yes)
Fourth ventricle compression (none/compressed/complete effacement)	IVH (no/yes)
Fourth ventricle extreme compression (none/completely effaced)	Cisternal tSAH (no/yes)
Lateral ventricle compression (none/any)	Cisternal tSAH completely filled (no/yes)
Lateral ventricle compression (none/mild/complete effacement)	Cisternal tSAH extent (none/visible/complete filling)
Tonsillar descent (no/yes)	Cortical tSAH extent (none/visible/> 90% cortical coverage)
Convexity SDH (no/yes)	Cortical tSAH thickness (none/< 5 mm/> 5 mm)
Tentorial SDH (no/yes)	DAI – BG (no/yes)
Falcine SDH (no/yes)	DAI – BS (no/yes)

*BG*, basal ganglia; *BS*, brain stem; *CC*, corpus callosum; *CT*, computed tomography; *DAI*, diffuse axonal injury; *EDH*, epidural hematoma; *IVH*, intra-ventricular hemorrhage; *MLS*, mid-line shift; *SC*, subcortical; *SDH*, subdural hematoma

## Results

### Patient demographics

As reported in the previous studies on this patient population, a total of 358 patients were included in the analysis. We focused only on both the first 48 h and first 10 days of physiologic recording for our signal analysis, allowing for direct comparison between admission CT imaging characteristics and cerebral compensatory reserve during the acute phase of ICU stay, as determine by RAP. The mean age was 40.6 ± 17.2 years, with 272 male patients. The median admission GCS was 7 (IQR, 3 to 9). The median 6-month GOS was 3 (range, 1 to 5). There was no difference in mean RAP variables between male and female patients, via the Mann *U* (*p* > 0.05 for all). Similarly, there was no correlation between RAP variables and patient age, with all the Pearson correlation coefficients and linear models between RAP variables and age being non-significant (i.e., all *r* ~ 0, with *p* > 0.05 for all).

### Association between RAP and admission CT characteristics—first 10 days of recording

All standard cranial CT scoring systems employed in TBI (Marshall [20], Rotterdam [18], Helsinki [23], and Stockholm [21]) failed to display a statistically significant correlation to RAP, regardless of the RAP variable tested (i.e., grand mean, percentage of time above threshold, and AUC over time). Similarly, all CT scoring systems failed to display a statistically significant increase in mean RAP variable, with increasing severity category.

**Fig. 2:**
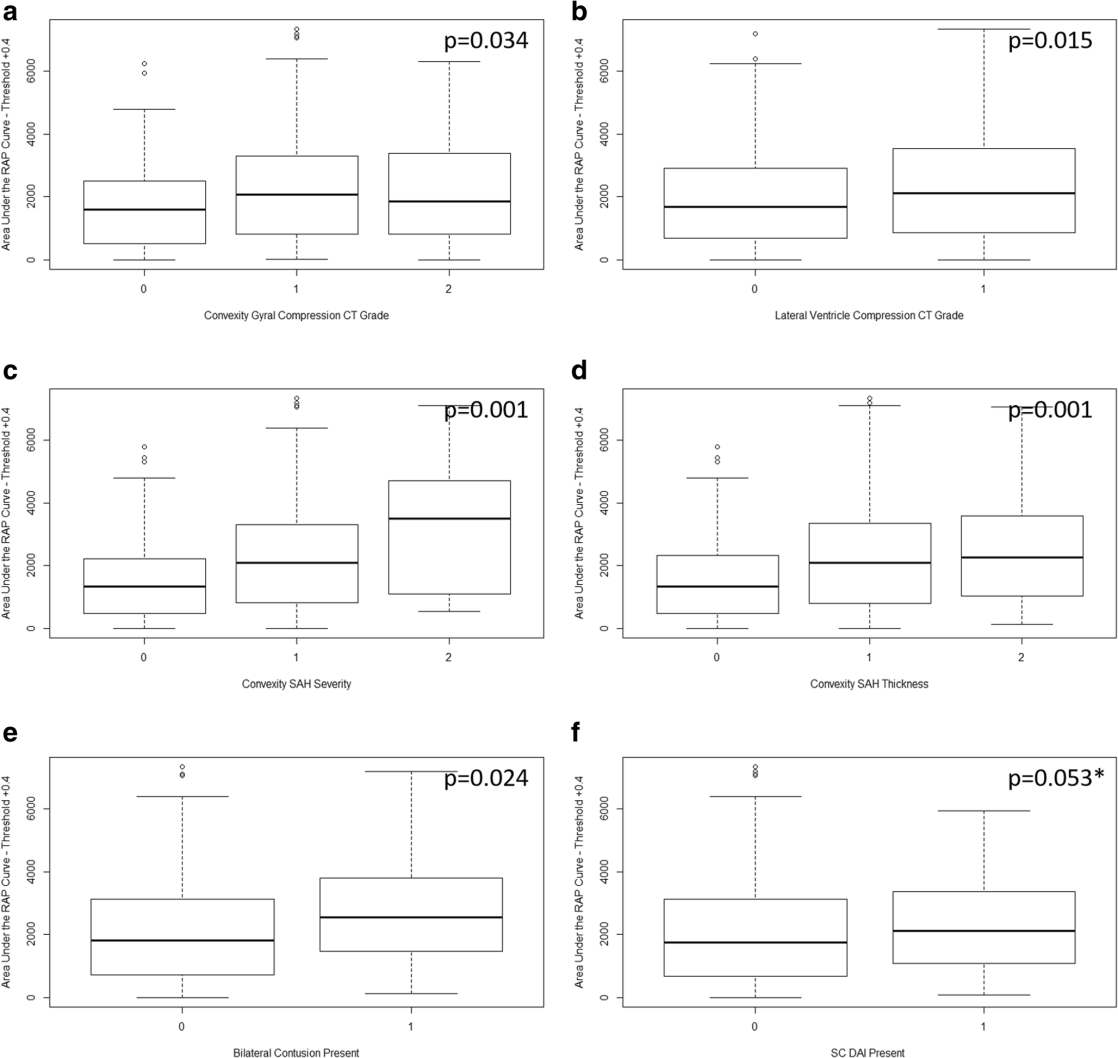
Box plots of AUC for RAP over time—first 10-day analysis, significantly associated imaging findings. **a** Mean RAP AUC vs ordinal convexity gyral compression score (0, none; 1, mild; 2, severe/complete effacement). **b** Mean RAP AUC vs ordinal lateral ventricle compression score (0, none; 1, any). **c** Mean RAP AUC vs cortical SAH severity score (0, none; 1, visible; 2, extensive bilateral location > 90% convexity). **d** Mean RAP AUC vs cortical SAH thickness score (0, none; 1 = < 5 mm; 2 = > 5 mm). **e** Mean RAP AUC vs presence of bilateral contusions (0, none; 1, present). **f** Mean RAP AUC vs presence of SC DAI (0, none; 1, present). All *p* values reported are for the Joncheere-Terpstra test, indicating statistically significant increase in mean RAP AUC with worsening injury score. *Of note, SC DAI was not significant for RAP AUC of 0.4, but was for AUC of 0.5, 0.6, and 0.7 (as seen in Appendix A). AUC, integrated area under the curve; CT, computed tomography; DAI, diffuse axonal injury; ICP, intra-cranial pressure; RAP, correlation between pulse amplitude of ICP (AMP) and ICP; SAH, subarachnoid hemorrhage; SC, subcortical. Above plots are for mean AUC for RAP over time using threshold of + 0.4 for RAP only. All other AUC thresholds tested can be seen in Appendix A

Individual admission CT characteristics which displayed a statistically significant association with RAP AUC over time (for RAP threshold of + 0.4) can be seen in Fig. 2, highlighting significant increase in mean values of the RAP variable with worsening injury score for the particular characteristic, using the Joncheere-Terpstra test. In general, AUC for RAP over time displayed significant increase for increases in the following ordinal CT characteristics: (a) cortical gyral effacement (0, no effacement; 1, partial effacement; 2, complete effacement), (b) lateral ventricle compression (0, none; 1, any), (c) bilateral contusions (0, absent, 1, present), (d) cortical subarachnoid hemorrhage (SAH) extent (0, none; 1, visible; 2, extensive bilaterally located > 90% of convexity), (e) cortical SAH thickness (0, none; 1, < 5 mm; 2, > 5 mm), and (f) subcortical DAI (0, absent; 1, present). This held true for most RAP AUC thresholds tested (i.e., AUC with RAP > 0, > + 0.2, > + 0.3, > + 0.4, > + 0.5, > + 0.6, and > +0.7). The results of the Joncheere-Terpstra tests for RAP AUC over time based on other thresholds (i.e., 0, + 0.2, + 0.3, + 0.5, + 0.6, and + 0.7) can be found in Appendix A of the supplementary materials.

Table 1 lists the admission CT characteristics that failed to be associated with any of the derived RAP variables during the first 10 days of recording. RAP grand mean and percentage of time over various RAP thresholds failed to reach consistent statistically significant relationships with any of the CT characteristics evaluated.

MLS, total contusion volume, and number of DAI lesions were not statistically associated with any of the RAP variables described, via the Pearson correlation coefficient and univariate linear modeling (*r* ~ 0 and *p* > 0.05 in all cases).

### Association between RAP and admission CT characteristics—first 48 h of recording

**Fig. 3:**
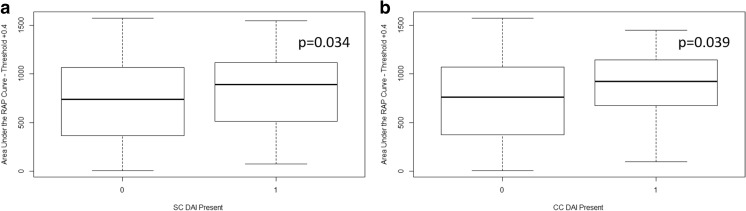
Box plots of AUC for RAP over time—first 48-h analysis, significantly associated imaging findings. **a** Mean RAP AUC vs presence of SC DAI (0, none; 1, present). **b** Mean RAP AUC vs presence of CC DAI (0, none; 1, present). All *p* values reported are for the Joncheere-Terpstra test, indicating statistically significant increase in mean RAP AUC with worsening injury score. AUC, integrated area under the curve; CC, corpus callosum; CT, computed tomography; DAI, diffuse axonal injury; ICP, intra-cranial pressure; RAP, correlation between pulse amplitude of ICP (AMP) and ICP; SC, subcortical. Above plots are for mean AUC for RAP over time using threshold of + 0.4 for RAP only. All other AUC thresholds tested can be seen in Appendix A

As with the first 10-day analysis, there were no statistically significant relationships seen between standard TBI CT scoring systems and the derived RAP variables. Overall, only two admission CT characteristics of diffuse cerebral injury were statistically associated with worse RAP AUC values, across most thresholds tested (AUC of RAP over time with thresholds of + 0.4, + 0.5, + 0.6, and + 0.7). The presence of subcortical and corpus callosal DAI lesions were statistically associated with higher RAP AUC values. Figure 3 highlights the significant increase in mean values of the RAP AUC over the thresholds of + 0.4 with worsening injury score for both subcortical and corpus callosal DAIs using the Joncheere-Terpstra test. The remainder of the significant results associated with these DAI lesions can be seen in Appendix B. This supports the associations found in the first 10-day analysis, though as mentioned in the “Methods” section, this analysis should be interpreted with great caution given our outlined database limitations.

Table 2 lists the admission CT characteristics that failed to be associated with any of the derived RAP variables during the first 48 h of recording. RAP grand mean and percentage of time over various RAP thresholds failed to reach consistent statistically significant relationships with any of the CT characteristics evaluated. All continuous CT variables tested failed to be associated with the RAP variables tested.

## Discussion

The results of this study provide for the first time an assessment of the association between the compensatory reserve index, RAP, and clinical relevant variables in adult TBI, admission cerebral injury characteristics. Some important points deserve highlighting.

First, standard cerebral CT grading schemes employed in TBI for outcome prediction are not predictive of impaired compensatory reserve, as measured by RAP over both the first 48 h and first 10 days of recording. This was the case for all derived RAP variables tested. This should not be a surprise, as these scoring systems were derived for the purpose of global outcome prediction in TBI, not the prediction of a physiologic event during the acute phase of illness. The lack of association between these scoring systems and RAP is mirrored by the lack of association seen between them and impaired cerebrovascular reactivity in TBI [28].

Second, admission cerebral CT imaging characteristics of diffuse injury/edema were statistically associated with worsening AUC of RAP over time. This was the case for both the first 48-h and first 10-day analysis. In particular, for the first 10-day analysis, the following injury patterns were found to be associated with worse compensatory reserve (i.e., higher AUC for RAP over time): cortical gyral effacement, lateral ventricle compression, bilateral contusions, presence of subcortical DAI, extent of cortical SAH, and the thickness of cortical SAH. All of these imaging characteristics are those of diffuse injury and/or edema, providing support that RAP is a measure of impaired compensatory reserve in adult TBI. For the first 48-h analysis, only the presence of subcortical or corpus callosal DAI was associated with statistically significant higher AUC of RAP over time. In both recording periods analyzed, these admission injury patterns are markers of diffuse acceleration/deceleration forces, which lead to cerebral edema and potentially impaired compensatory reserve, as seen with the worsening AUC for RAP over time. Furthermore, these injury patterns are also similar to those identified to be associated with impaired cerebrovascular reactivity [28]. Thus, the potential link exists between worsening cerebrovascular reactivity and subsequent impaired compensatory reserve, as seen in the recent work describing the combined index RAC (correlation between AMP and CPP), though this requires further investigation. One major limitation of this current study is the lack of sequential follow-up CT scans to assess if these imaging characteristics are worsening as the AUC of RAP over time becomes larger, another area for future research.

Finally, various admission CT findings consisted with gross macroscopic parenchymal injury appear to not be association with RAP during the course of ICU monitoring. This held true regardless of the CT injury characteristic tested in both the first 48-h and first 10-day analysis. In particular, midline shift and contusion volume failed to demonstrate a significant link to RAP. This is similar to the results seen for PRx, PAx, and RAC [28].

### Limitations

Despite the interesting preliminary results, some important limitations should be emphasized. First, this is a retrospective cohort analysis. The patient’s physiologic data was collected over a long period of time, 2005 to 2016. Limited patient demographics were available from our database, with only age, sex, and admission total GCS available. No information was available for GCS motor score or admission pupillary status. Furthermore, despite having standard treatments for ICP and CPP within the NCCU at the Addenbrooke’s, there exists the potential for treatment heterogeneity between patients, which could have impacted the physiologic signals recorded, and thus the results of the described analysis. An important aspect related to this is the timing of the recorded signals in relation to the injury. As mentioned within the “Methods” section, the database does not record date or time of injury, or the delay between injury and ICP monitor placement/signal recording. This is a major limitation of the study. Most patients admitted to the NCCU have their monitors placed within the first 24 to 48 h. However, there are patients, based on clinical condition, which are admitted for clinical and radiologic monitoring with invasive ICP monitors placed at dates later than 48 h post-injury (even up to days post-injury). Thus, the majority of our analysis reported in the manuscript is based on the first 10 days of recording, not the first 10 days of physiology post-injury. The same limitation holds true for the first 48-h analysis that has been briefly described. It would have been more ideal to accurately focus on a definitive time frame of recorded physiology closer to the injury and admission CT scan, such as the first 24 or 48 h after injury, allowing for a more definitive assessment of the relation between imaging markers of injury and cerebral compensatory reserve. Unfortunately, the information allowing for this analysis is not available and cannot be retrieved. As such, the analysis described above is only preliminary and requires much further validation using prospectively collected data sets. This more detailed temporal analysis is planned using the multi-center CENTER-TBI high-resolution data set [19].

Second, our method of analyzing the association between AUC over time for RAP and its association with CT markers can be questioned. As this is a new index in clinical TBI research, it currently remains unknown as to the best approach for handling the data from RAP. One could argue that AUC over the first 48 h or first 10 days of the recording period may not be the best approach. Perhaps hourly dosing of RAP above particular threshold may be superior. This has yet to be shown. Given the limitations related to timing of injury and timing of physiologic recording, mentioned above, it is impossible with the current database to definitively assess the temporal correlation between injury and cerebral physiology immediately after the insult. As the work presented in this manuscript consists of the first ever description of the association between this continuous index of compensatory reserve and admission CT findings of diffuse injury, it should be considered preliminary and experimental. At the current time, we in no way suggest that this index should be used clinically to guide therapy. Much further work is required to determine the optimal method for analyzing such data derived from RAP and provide validation for the results found in this paper, not to mention assess the temporal relation between this measure and sequential follow-up neuroimaging. All of this analysis is planned with local retrospective experimental model data, in addition to the available multi-center prospective TBI data collected as part of CENTER-TBI [19]. Our current work provides only a snapshot of the potential for this index, with an immense amount of further work required.

Third, we must re-emphasize that any analysis based on our retrospective signal database is severely limited due to inconsistent time of recording onset. In particular, any results (positive or negative) from the first 48-h analysis are extremely difficult to interpret as the onset to signal recording post-injury is quite variable. A lack of significant findings with respect to specific CT injury characteristics of diffuse injury or elevated ICP on such an analysis does not disprove that RAP is a measure of compensatory reserve during the “early” post-injury phase. Further, significant results during that time are also questionable given the data limitations. This is why we have focused the majority of the reporting on the “acute” ICU phase of care (i.e., first 10 days of recording). Again, the current analysis provided is just the preliminary work in this area. Much further work is required to confirm relationships seen in our manuscript, including a more directed and reliable temporal analysis, in conjunction with serial imaging analysis.

Fourth, only preliminary evidence exists to suggest the added value of RAP monitoring over ICP monitoring alone [4, 22]. This current analysis was not designed to address whether RAP provides additional value over ICP monitoring alone; it was only directed towards providing some preliminary evidence to suggest RAP is indeed a marker of intra-cranial compensatory reserve, as assessed via admission CT-based injury markers. The previous work suggests that the addition of RAP monitoring to ICP, in the setting of adult TBI, does provide outcome predictive capability [4]. Though it must be acknowledged that this work is a single center retrospective analysis, much further work, using multi-variable analytic techniques, are required to determine the exact added value of RAP monitoring to ICP alone, as it pertains to patient outcome and imaging-based lesion change/progression. This analysis is planned utilizing the multi-center TBI patient cohort from CENTER-TBI [19].

Finally, the imaging characteristics were only available for admission CT scans. Thus, only associations between these characteristics and RAP could be made. We were unable to comment on the association between CT changes over time and RAP. This is an area for future research, as demonstrating the link between worsening RAP and progression of injury on CT may provide a useful application for continuous monitoring of this index within the ICU. The high-resolution data set from CENTER-TBI [19] may be best positioned to answer this question, using automated quantifiable assessments of sequential CT scans within this population of critically ill TBI patients.

## Conclusions

RAP is associated with admission cerebral CT injury patterns of diffuse injury and edema, providing some confirmation of its potential measurement of cerebral compensatory reserve in TBI.

## Electronic supplementary material

ESM 1 (DOCX 24 kb)

ESM 2 (DOCX 16 kb)
