# Supplementary material for: Impaired cerebral compensatory reserve is associated with admission imaging characteristics of diffuse insult in traumatic brain injury
Source: Acta Neurochir (Wien). 2018 Sep 24;160(12):2277–87. doi: 10.1007/s00701-018-3681-y (PMC6267721; doi:10.1007/s00701-018-3681-y)
Supplement: Supplementary file 1 — (DOCX 24 kb) [file 701_2018_3681_MOESM1_ESM.docx]

**Appendix A: Joncheere-Terpstra Test for RAP AUC Using Other Thresholds – 1^st^ 10 Days of Recording Analysis**

***Note: Below are the Joncheere-Terpstra test results for the other RAP AUC thresholds tested: 0, +0.2, +0.3, +0.5, +0.6, +0.7; for each of the significant admission CT injury characteristics. A p<0.05 indicates that there is a statistically significant increase in the mean RAP AUC value with progressive increase in ordinal injury category.**

1. **Convexity Gyral Compression (0 = none, 1 = mild, 2 = severe/complete effacement)**

**RAP AUC with RAP Threshold of 0**

> jonckheere.test(final$AUC_0,final$'Convexity Gyri (Normal = 0/mild compression = 1/compressed-absent = 2)',alternative="increasing",nperm=1000)

Jonckheere-Terpstra test

data:

JT = 22651, p-value = 0.019

alternative hypothesis: increasing

**RAP AUC with RAP Threshold of 0.2**

> jonckheere.test(final$AUC_0.2,final$'Convexity Gyri (Normal = 0/mild compression = 1/compressed-absent = 2)',alternative="increasing",nperm=1000)

Jonckheere-Terpstra test

data:

JT = 22575, p-value = 0.02

alternative hypothesis: increasing

**RAP AUC with RAP Threshold of 0.3**

> jonckheere.test(final$AUC_0.3,final$'Convexity Gyri (Normal = 0/mild compression = 1/compressed-absent = 2)',alternative="increasing",nperm=1000)

Jonckheere-Terpstra test

data:

JT = 22491, p-value = 0.032

alternative hypothesis: increasing

**RAP AUC with RAP Threshold of 0.4**

> jonckheere.test(final$AUC_0.4,final$'Convexity Gyri (Normal = 0/mild compression = 1/compressed-absent = 2)',alternative="increasing",nperm=1000)

Jonckheere-Terpstra test

data:

JT = 22413, p-value = 0.034

alternative hypothesis: increasing

**RAP AUC with RAP Threshold of 0.5**

> jonckheere.test(final$AUC_0.5,final$'Convexity Gyri (Normal = 0/mild compression = 1/compressed-absent = 2)',alternative="increasing",nperm=1000)

Jonckheere-Terpstra test

data:

JT = 22258, p-value = 0.053

alternative hypothesis: increasing

**RAP AUC with RAP Threshold of 0.6**

> jonckheere.test(final$AUC_0.6,final$'Convexity Gyri (Normal = 0/mild compression = 1/compressed-absent = 2)',alternative="increasing",nperm=1000)

Jonckheere-Terpstra test

data:

JT = 22128, p-value = 0.051

alternative hypothesis: increasing

**RAP AUC with RAP Threshold of 0.7**

> jonckheere.test(final$AUC_0.7,final$'Convexity Gyri (Normal = 0/mild compression = 1/compressed-absent = 2)',alternative="increasing",nperm=1000)

Jonckheere-Terpstra test

data:

JT = 21954, p-value = 0.107

alternative hypothesis: increasing

1. **Lateral Ventricle Compression (0 = none, 1 = any)**

**RAP AUC with RAP Threshold of 0**

> jonckheere.test(final$AUC_0,final$'Lat Vent (any comp = 1, none = 0)',alternative="increasing",nperm=1000)

Jonckheere-Terpstra test

data:

JT = 17956, p-value = 0.006

alternative hypothesis: increasing

**RAP AUC with RAP Threshold of 0.2**

> jonckheere.test(final$AUC_0.2,final$'Lat Vent (any comp = 1, none = 0)',alternative="increasing",nperm=1000)

Jonckheere-Terpstra test

data:

JT = 17855, p-value = 0.01

alternative hypothesis: increasing

**RAP AUC with RAP Threshold of 0.3**

> jonckheere.test(final$AUC_0.3,final$'Lat Vent (any comp = 1, none = 0)',alternative="increasing",nperm=1000)

Jonckheere-Terpstra test

data:

JT = 17775, p-value = 0.013

alternative hypothesis: increasing

**RAP AUC with RAP Threshold of 0.4**

> jonckheere.test(final$AUC_0.4,final$'Lat Vent (any comp = 1, none = 0)',alternative="increasing",nperm=1000)

Jonckheere-Terpstra test

data:

JT = 17728, p-value = 0.015

alternative hypothesis: increasing

**RAP AUC with RAP Threshold of 0.5**

> jonckheere.test(final$AUC_0.5,final$'Lat Vent (any comp = 1, none = 0)',alternative="increasing",nperm=1000)

Jonckheere-Terpstra test

data:

JT = 17613, p-value = 0.017

alternative hypothesis: increasing

**RAP AUC with RAP Threshold of 0.6**

> jonckheere.test(final$AUC_0.6,final$'Lat Vent (any comp = 1, none = 0)',alternative="increasing",nperm=1000)

Jonckheere-Terpstra test

data:

JT = 17504, p-value = 0.027

alternative hypothesis: increasing

**RAP AUC with RAP Threshold of 0.7**

> jonckheere.test(final$AUC_0.7,final$'Lat Vent (any comp = 1, none = 0)',alternative="increasing",nperm=1000)

Jonckheere-Terpstra test

data:

JT = 17344, p-value = 0.035

alternative hypothesis: increasing

1. **Convexity SAH Extent (0 = non, 1 = some present, 2 = >90% convexity coverage)**

**RAP AUC with RAP Threshold of 0**

> jonckheere.test(final$AUC_0,final$'tSAH-conv (Extensive bilat (>90% convexity) = 2/visible in gyri = 1/ Absent = 0)',alternative="increasing",nperm=1000)

Jonckheere-Terpstra test

data:

JT = 16920, p-value = 0.002

alternative hypothesis: increasing

**RAP AUC with RAP Threshold of 0.2**

> jonckheere.test(final$AUC_0.2,final$'tSAH-conv (Extensive bilat (>90% convexity) = 2/visible in gyri = 1/ Absent = 0)',alternative="increasing",nperm=1000)

Jonckheere-Terpstra test

data:

JT = 16861, p-value = 0.001

alternative hypothesis: increasing

**RAP AUC with RAP Threshold of 0.3**

> jonckheere.test(final$AUC_0.3,final$'tSAH-conv (Extensive bilat (>90% convexity) = 2/visible in gyri = 1/ Absent = 0)',alternative="increasing",nperm=1000)

Jonckheere-Terpstra test

data:

JT = 16782, p-value = 0.002

alternative hypothesis: increasing

**RAP AUC with RAP Threshold of 0.4**

> jonckheere.test(final$AUC_0.4,final$'tSAH-conv (Extensive bilat (>90% convexity) = 2/visible in gyri = 1/ Absent = 0)',alternative="increasing",nperm=1000)

Jonckheere-Terpstra test

data:

JT = 16711, p-value = 0.001

alternative hypothesis: increasing

**RAP AUC with RAP Threshold of 0.5**

> jonckheere.test(final$AUC_0.5,final$'tSAH-conv (Extensive bilat (>90% convexity) = 2/visible in gyri = 1/ Absent = 0)',alternative="increasing",nperm=1000)

Jonckheere-Terpstra test

data:

JT = 16607, p-value = 0.001

alternative hypothesis: increasing

**RAP AUC with RAP Threshold of 0.6**

> jonckheere.test(final$AUC_0.6,final$'tSAH-conv (Extensive bilat (>90% convexity) = 2/visible in gyri = 1/ Absent = 0)',alternative="increasing",nperm=1000)

Jonckheere-Terpstra test

data:

JT = 16484, p-value = 0.001

alternative hypothesis: increasing

**RAP AUC with RAP Threshold of 0.7**

> jonckheere.test(final$AUC_0.7,final$'tSAH-conv (Extensive bilat (>90% convexity) = 2/visible in gyri = 1/ Absent = 0)',alternative="increasing",nperm=1000)

Jonckheere-Terpstra test

data:

JT = 16271, p-value = 0.001

alternative hypothesis: increasing

1. **Convexity SAH Thickness (0 = none, 1 = <5mm, 2 = >5mm)**

**RAP AUC with RAP Threshold of 0**

> jonckheere.test(final$AUC_0,final$'tSAH-conv thickness (>5mm = 2/1-5mm = 1/no blood = 0; *Note: visible blood is at least 1mm)',alternative="increasing",nperm=1000)

Jonckheere-Terpstra test

data:

JT = 20646, p-value = 0.001

alternative hypothesis: increasing

**RAP AUC with RAP Threshold of 0.2**

> jonckheere.test(final$AUC_0.2,final$'tSAH-conv thickness (>5mm = 2/1-5mm = 1/no blood = 0; *Note: visible blood is at least 1mm)',alternative="increasing",nperm=1000)

Jonckheere-Terpstra test

data:

JT = 20665, p-value = 0.001

alternative hypothesis: increasing

**RAP AUC with RAP Threshold of 0.3**

> jonckheere.test(final$AUC_0.3,final$'tSAH-conv thickness (>5mm = 2/1-5mm = 1/no blood = 0; *Note: visible blood is at least 1mm)',alternative="increasing",nperm=1000)

Jonckheere-Terpstra test

data:

JT = 20628, p-value = 0.002

alternative hypothesis: increasing

**RAP AUC with RAP Threshold of 0.4**

> jonckheere.test(final$AUC_0.4,final$'tSAH-conv thickness (>5mm = 2/1-5mm = 1/no blood = 0; *Note: visible blood is at least 1mm)',alternative="increasing",nperm=1000)

Jonckheere-Terpstra test

data:

JT = 20606, p-value = 0.001

alternative hypothesis: increasing

**RAP AUC with RAP Threshold of 0.5**

> jonckheere.test(final$AUC_0.5,final$'tSAH-conv thickness (>5mm = 2/1-5mm = 1/no blood = 0; *Note: visible blood is at least 1mm)',alternative="increasing",nperm=1000)

Jonckheere-Terpstra test

data:

JT = 20551, p-value = 0.001

alternative hypothesis: increasing

**RAP AUC with RAP Threshold of 0.6**

> jonckheere.test(final$AUC_0.6,final$'tSAH-conv thickness (>5mm = 2/1-5mm = 1/no blood = 0; *Note: visible blood is at least 1mm)',alternative="increasing",nperm=1000)

Jonckheere-Terpstra test

data:

JT = 20471, p-value = 0.001

alternative hypothesis: increasing

**RAP AUC with RAP Threshold of 0.7**

> jonckheere.test(final$AUC_0.7,final$'tSAH-conv thickness (>5mm = 2/1-5mm = 1/no blood = 0; *Note: visible blood is at least 1mm)',alternative="increasing",nperm=1000)

Jonckheere-Terpstra test

data:

JT = 20255, p-value = 0.001

alternative hypothesis: increasing

1. **Bilateral Contusion (0 = none, 1 = present)**

**RAP AUC with RAP Threshold of 0**

> jonckheere.test(final$AUC_0,final$'Bilat ICH-contusion (Absent = 0/Present = 1)',alternative="increasing",nperm=1000)

Jonckheere-Terpstra test

data:

JT = 6926, p-value = 0.012

alternative hypothesis: increasing

**RAP AUC with RAP Threshold of 0.2**

> jonckheere.test(final$AUC_0.2,final$'Bilat ICH-contusion (Absent = 0/Present = 1)',alternative="increasing",nperm=1000)

Jonckheere-Terpstra test

data:

JT = 6879, p-value = 0.011

alternative hypothesis: increasing

**RAP AUC with RAP Threshold of 0.3**

> jonckheere.test(final$AUC_0.3,final$'Bilat ICH-contusion (Absent = 0/Present = 1)',alternative="increasing",nperm=1000)

Jonckheere-Terpstra test

data:

JT = 6842, p-value = 0.026

alternative hypothesis: increasing

**RAP AUC with RAP Threshold of 0.4**

> jonckheere.test(final$AUC_0.4,final$'Bilat ICH-contusion (Absent = 0/Present = 1)',alternative="increasing",nperm=1000)

Jonckheere-Terpstra test

data:

JT = 6783, p-value = 0.024

alternative hypothesis: increasing

**RAP AUC with RAP Threshold of 0.5**

> jonckheere.test(final$AUC_0.5,final$'Bilat ICH-contusion (Absent = 0/Present = 1)',alternative="increasing",nperm=1000)

Jonckheere-Terpstra test

data:

JT = 6727, p-value = 0.03

alternative hypothesis: increasing

**RAP AUC with RAP Threshold of 0.6**

> jonckheere.test(final$AUC_0.6,final$'Bilat ICH-contusion (Absent = 0/Present = 1)',alternative="increasing",nperm=1000)

Jonckheere-Terpstra test

data:

JT = 6654, p-value = 0.041

alternative hypothesis: increasing

**RAP AUC with RAP Threshold of 0.7**

> jonckheere.test(final$AUC_0.7,final$'Bilat ICH-contusion (Absent = 0/Present = 1)',alternative="increasing",nperm=1000)

Jonckheere-Terpstra test

data:

JT = 6490, p-value = 0.051

alternative hypothesis: increasing

1. **DAI – Sub-Cortical**

**RAP AUC with RAP Threshold of 0**

> jonckheere.test(final$AUC_0,final$'DAI-SC (Present = 1/Absent = 0)',alternative="increasing",nperm=1000)

Jonckheere-Terpstra test

data:

JT = 12333, p-value = 0.08

alternative hypothesis: increasing

**RAP AUC with RAP Threshold of 0.2**

> jonckheere.test(final$AUC_0.2,final$'DAI-SC (Present = 1/Absent = 0)',alternative="increasing",nperm=1000)

Jonckheere-Terpstra test

data:

JT = 12436, p-value = 0.067

alternative hypothesis: increasing

**RAP AUC with RAP Threshold of 0.3**

> jonckheere.test(final$AUC_0.3,final$'DAI-SC (Present = 1/Absent = 0)',alternative="increasing",nperm=1000)

Jonckheere-Terpstra test

data:

JT = 12482, p-value = 0.054

alternative hypothesis: increasing

**RAP AUC with RAP Threshold of 0.4**

> jonckheere.test(final$AUC_0.4,final$'DAI-SC (Present = 1/Absent = 0)',alternative="increasing",nperm=1000)

Jonckheere-Terpstra test

data:

JT = 12577, p-value = 0.053

alternative hypothesis: increasing

**RAP AUC with RAP Threshold of 0.5**

> jonckheere.test(final$AUC_0.5,final$'DAI-SC (Present = 1/Absent = 0)',alternative="increasing",nperm=1000)

Jonckheere-Terpstra test

data:

JT = 12645, p-value = 0.044

alternative hypothesis: increasing

**RAP AUC with RAP Threshold of 0.6**

> jonckheere.test(final$AUC_0.6,final$'DAI-SC (Present = 1/Absent = 0)',alternative="increasing",nperm=1000)

Jonckheere-Terpstra test

data:

JT = 12764, p-value = 0.016

alternative hypothesis: increasing

**RAP AUC with RAP Threshold of 0.7**

> jonckheere.test(final$AUC_0.7,final$'DAI-SC (Present = 1/Absent = 0)',alternative="increasing",nperm=1000)

Jonckheere-Terpstra test

data:

JT = 12907, p-value = 0.015

alternative hypothesis: increasing
