# Supplementary material for: Impaired cerebral compensatory reserve is associated with admission imaging characteristics of diffuse insult in traumatic brain injury
Source: Acta Neurochir (Wien). 2018 Sep 24;160(12):2277–87. doi: 10.1007/s00701-018-3681-y (PMC6267721; doi:10.1007/s00701-018-3681-y)
Supplement: Supplementary file 2 — (DOCX 16 kb) [file 701_2018_3681_MOESM2_ESM.docx]

**Appendix B: Joncheere-Terpstra Test for RAP AUC Using Other Thresholds – 1^st^ 48 Hours of Recording Analysis**

***Note: Below are the Joncheere-Terpstra test results for the other RAP AUC thresholds tested: 0, +0.2, +0.3, +0.5, +0.6, +0.7; for each of the significant admission CT injury characteristics. A p<0.05 indicates that there is a statistically significant increase in the mean RAP AUC value with progressive increase in ordinal injury category.**

1. **SC DAI (0 = Absent, 1 = Present)**

**RAP AUC with RAP Threshold of 0**

> jonckheere.test(final$AUC_0,final$'DAI-SC (Present = 1/Absent = 0)',alternative="increasing",nperm=1000)

Jonckheere-Terpstra test

data:

JT = 12400, p-value = 0.081

alternative hypothesis: increasing

>

**RAP AUC with RAP Threshold of 0.2**

> jonckheere.test(final$AUC_0.2,final$'DAI-SC (Present = 1/Absent = 0)',alternative="increasing",nperm=1000)

Jonckheere-Terpstra test

data:

JT = 12490, p-value = 0.067

alternative hypothesis: increasing

>

**RAP AUC with RAP Threshold of 0.3**

> jonckheere.test(final$AUC_0.3,final$'DAI-SC (Present = 1/Absent = 0)',alternative="increasing",nperm=1000)

Jonckheere-Terpstra test

data:

JT = 12561, p-value = 0.067

alternative hypothesis: increasing

>

**RAP AUC with RAP Threshold of 0.4**

> jonckheere.test(final$AUC_0.4,final$'DAI-SC (Present = 1/Absent = 0)',alternative="increasing",nperm=1000)

Jonckheere-Terpstra test

data:

JT = 12630, p-value = 0.034

alternative hypothesis: increasing

>

**RAP AUC with RAP Threshold of 0.5**

> jonckheere.test(final$AUC_0.5,final$'DAI-SC (Present = 1/Absent = 0)',alternative="increasing",nperm=1000)

Jonckheere-Terpstra test

data:

JT = 12715, p-value = 0.018

alternative hypothesis: increasing

>

**RAP AUC with RAP Threshold of 0.6**

> jonckheere.test(final$AUC_0.6,final$'DAI-SC (Present = 1/Absent = 0)',alternative="increasing",nperm=1000)

Jonckheere-Terpstra test

data:

JT = 12822, p-value = 0.025

alternative hypothesis: increasing

>

**RAP AUC with RAP Threshold of 0.7**

> jonckheere.test(final$AUC_0.7,final$'DAI-SC (Present = 1/Absent = 0)',alternative="increasing",nperm=1000)

Jonckheere-Terpstra test

data:

JT = 12904, p-value = 0.027

alternative hypothesis: increasing

**2. CC DAI (0 = Absent, 1 = Present)**

**RAP AUC with RAP Threshold of 0**

jonckheere.test(final$AUC_0,final$'DAI-CC (Present = 1/Absent = 0)',alternative="increasing",nperm=1000)

Jonckheere-Terpstra test

data:

JT = 4192, p-value = 0.064

alternative hypothesis: increasing

>

**RAP AUC with RAP Threshold of 0.2**

> jonckheere.test(final$AUC_0.2,final$'DAI-CC (Present = 1/Absent = 0)',alternative="increasing",nperm=1000)

Jonckheere-Terpstra test

data:

JT = 4225, p-value = 0.069

alternative hypothesis: increasing

>

**RAP AUC with RAP Threshold of 0.3**

> jonckheere.test(final$AUC_0.3,final$'DAI-CC (Present = 1/Absent = 0)',alternative="increasing",nperm=1000)

Jonckheere-Terpstra test

data:

JT = 4253, p-value = 0.049

alternative hypothesis: increasing

>

**RAP AUC with RAP Threshold of 0.4**

> jonckheere.test(final$AUC_0.4,final$'DAI-CC (Present = 1/Absent = 0)',alternative="increasing",nperm=1000)

Jonckheere-Terpstra test

data:

JT = 4270, p-value = 0.039

alternative hypothesis: increasing

>

**RAP AUC with RAP Threshold of 0.5**

> jonckheere.test(final$AUC_0.5,final$'DAI-CC (Present = 1/Absent = 0)',alternative="increasing",nperm=1000)

Jonckheere-Terpstra test

data:

JT = 4276, p-value = 0.052

alternative hypothesis: increasing

>

**RAP AUC with RAP Threshold of 0.6**

> jonckheere.test(final$AUC_0.6,final$'DAI-CC (Present = 1/Absent = 0)',alternative="increasing",nperm=1000)

Jonckheere-Terpstra test

data:

JT = 4313, p-value = 0.045

alternative hypothesis: increasing

>

**RAP AUC with RAP Threshold of 0.7**

> jonckheere.test(final$AUC_0.7,final$'DAI-CC (Present = 1/Absent = 0)',alternative="increasing",nperm=1000)

Jonckheere-Terpstra test

data:

JT = 4343, p-value = 0.034

alternative hypothesis: increasing
